# The Histone Deacetylase HstD Regulates Fungal Growth, Development and Secondary Metabolite Biosynthesis in *Aspergillus terreus*

**DOI:** 10.3390/ijms241612569

**Published:** 2023-08-08

**Authors:** Guangshan Yao, Na Han, Huawei Zheng, Lu Wang

**Affiliations:** 1 Fujian Key Laboratory on Conservation and Sustainable Utilization of Marine Biodiversity, Institute of Oceanography, Minjiang University, Fuzhou 350108, Chinana17076428485@163.com (N.H.); zhw@mju.edu.cn (H.Z.); 2 Key Laboratory of Marine Drugs, The Ministry of Education of China, School of Medicine and Pharmacy, Ocean University of China, Qingdao 266003, China

**Keywords:** *Aspergillus terreus*, deacetylase, secondary metabolism, lovastatin, terrein

## Abstract

Histone acetylation modification significantly affects secondary metabolism in filamentous fungi. However, how histone acetylation regulates secondary metabolite synthesis in the lovastatin (a lipid-lowering drug) producing *Aspergillus terreus* remains unknown because protein is involved and has been identified in this species. Here, the fungal-specific histone deacetylase gene, *hstD*, was characterized through functional genomics in two marine-derived *A. terreus* strains, Mj106 and RA2905. The results showed that the ablation of HstD resulted in reduced mycelium growth, less conidiation, and decreased lovastatin biosynthesis but significantly increased terrein biosynthesis. However, unlike its homologs in yeast, HstD was not required for fungal responses to DNA damage agents, indicating that HstD likely plays a novel role in the DNA damage repair process in *A. terreus*. Furthermore, the loss of HstD resulted in a significant upregulation of H3K56 and H3K27 acetylation when compared to the wild type, suggesting that epigenetic functions of HstD, as a deacetylase, target H3K27 and H3K56. Additionally, a set of no-histone targets with potential roles in fungal growth, conidiation, and secondary metabolism were identified for the first time using acetylated proteomic analysis. In conclusion, we provide a comprehensive analysis of HstD for its targets in histone or non-histone and its roles in fungal growth and development, DNA damage response, and secondary metabolism in *A. terreus*.

## 1. Introduction

The filamentous fungus *Aspergillus terreus* is an outstanding producer of various bioactive secondary metabolites. One of the most valued and well-known secondary metabolites is the cholesterol-lowering drug lovastatin, which competitively inhibits the rate-limiting enzyme HMG-CoA reductase in the cholesterol synthesis pathway and has long been approved for the treatment of hypercholesterolemia [1]. Terrein, another polyketide compound produced by *A. terreus*, inhibits the proliferation of both keratinocyte and hepatoma cells by blocking cell cycle gene expression [2,3,4]. Another secondary metabolite, citreoviridin, acts as an inhibitor of ATP synthase and has been reported to treat breast cancer in combination therapy [5]. Aspterric acid, a sesquiterpene from *A. terreus*, has been demonstrated to be a specific inhibitor of dihydroxy acid dehydratase, showing great promise as a biosafe herbicide [6]. Recently, a variety of new secondary metabolites have been identified, including cytotoxic emestrins L and M, anti-microbial (+)- and (−)-asperfuranone, and asperpyranones A and B [7,8]. However, because the regulatory mechanism for secondary metabolism is not fully understood, the production of these valuable compounds remains at a very low level.

Histone acetylation participates in many cellular processes in filamentous fungi, including morphogenesis, conidiation, stress response, and secondary metabolism [9]. The acetylation level of histones is controlled by histone acetyltransferases and deacetylases, which is a dynamic process. Histone deacetylases are a class of enzymes that catalyze the deacetylation of lysine at the N-terminal of histone and other non-histone proteins [10]. Recently, the regulation of secondary metabolism by histone deacetylases has been widely reported in filamentous fungi. In *A. nidulans*, the major histone deacetylase HdaA specifically regulates the expression of two subtelomeric gene clusters for the synthesis of sterigmatocystin and penicillium, and its deletion results in an increase in the production of these secondary metabolites [11]. In *A. flavus*, the sirtuin histone deacetylase SirE was reported to regulate fungal development, DNA damage response, and aflatoxin production [12]. In the endophytic fungus *Penicillium chrysogenum* Fes1701, the deletion of the *hdaA* gene induced a significant and complex change in the secondary metabolite profile [13]. The chemical inhibition of histone deacetylases has been an efficient strategy to activate the silent gene cluster for secondary metabolites in multiple fungi [10]. In this way, new secondary metabolites were identified in a marine-derived *A. terreus* RA2905 [8]. However, the molecular mechanism for the regulation of secondary metabolism by histone acetylation is unexplored, and histone acetyltransferases or deacetylases are less well identified in *A. terreus*.

In the present study, the fungal-specific histone deacetylase was identified and characterized in two *A. terreus* strains with different secondary metabolite profiles. The roles of *hstD* in fungal morphology, conidiation, DNA stress response, and the biosynthesis of secondary metabolites were investigated by gene-loss-function analysis. The histone acetylation level at H3K56 was analyzed in WT and *hstD* gene deletion mutants. Novel non-histone targets of HstD in *A. terreus* were explored using acetylation proteomic analysis.

## 2. Results

### 2.1. Bioinformatic Analysis of Sirtuin Deacetylases

Our previous results demonstrated that the metabolite profile of *A. terreus* could be significantly altered by a chemical epigenetic modification using a histone deacetylase inhibitor, suggesting that histone deacetylases have regulatory roles in the secondary metabolism of *A. terreus* [8]. Among histone deacetylases, sirtuin family proteins have been shown to affect fungal morphogenesis, development, and secondary metabolites in various model and pathogenic fungi [14] but have not been explored in *A. terreus*. Using known sirtuins, including HST1, HST3, or HST4, from *Saccharomyces cerevisiae* or *A. nidulans* as baits, a search was conducted against the *A. terreus* genome database, and a total of six putative sirtuin deacetylases were manually confirmed by suing InterProScan on the EBI web server. 

**Figure 1:**
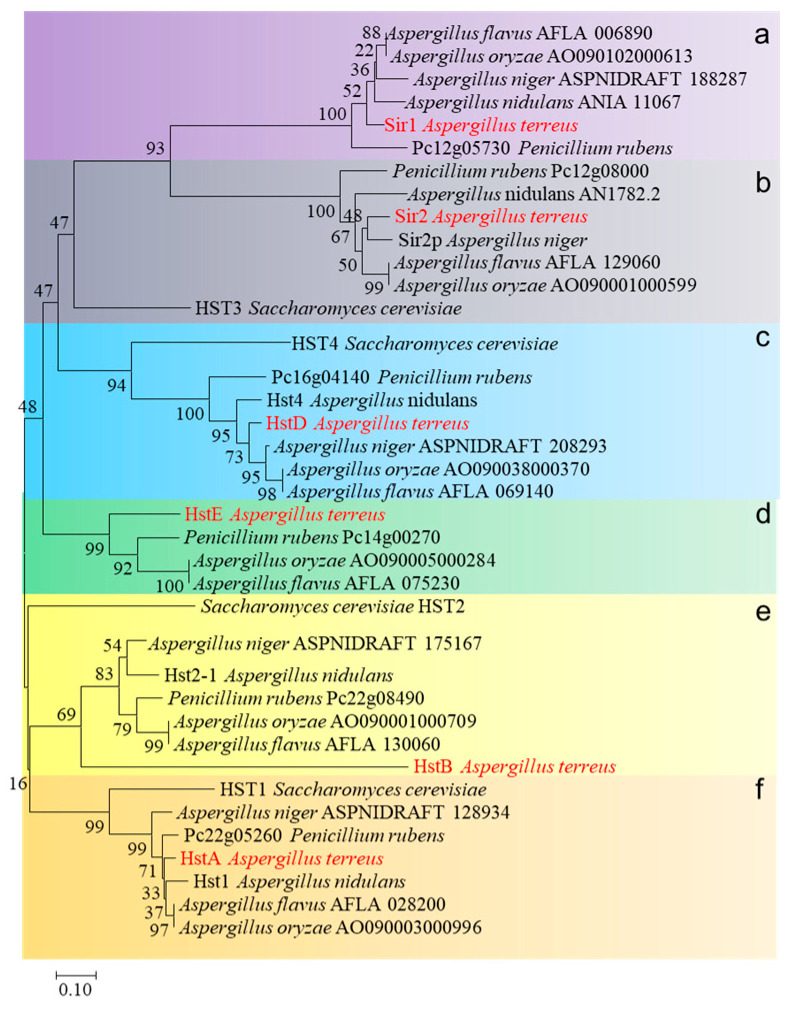
The phylogenetic tree was constructed by comparing amino acid sequences among different species using the neighbor-joining algorithm via MEGA6.

Subsequently, a phylogenetic analysis of sirtuins from *S. cerevisiae, A. nidulans, A. flavus, A. oxyzae, A. niger, Penicillium rubens*, and *A. terreus* was performed. These 38 sirtuins were divided into six clades (from clades a–f, Figure 1). Sirtuins from filamentous fungi were evenly classified into each clade: only one sirtuin named HST3 (yeast) was shared by both clades a and b, and no yeast homologs were identified in clades d and e. These results suggest that sirtuins underwent gene expansion in filamentous fungi, leading to a divergence between yeast and multicellular filamentous fungi. Interestingly, among these sirtuin deacetylases, sirtuins of clade c uniquely exist in the genome of the fungi and not in higher eukaryotic organisms. Compared to clade c, other clade deacetylases were widely dissected in various model organisms. For example, clade b (Sir2) involves caloric restriction and aging and is highly conserved from bacteria to humans [15]. Clade a (Sir1) is pivotal in causing heterochromatin to assemble at and spread from silencers [16]. Sir2 or Sir1 seem to have no role in fungal-specific processes, such as conidiation or secondary metabolism in fungi. Deacetylases from clade c were identified in *Monascus ruber* and *A. nidulans*; these data consistently implied that HstD (Hst4) was specifically involved in fungal growth, development, or secondary metabolism, suggesting a gain-of-function during the gene expansion of sirtuin. Therefore, HstD from clade c was the focus of this study, and its specific roles in fungal growth, development, stress response to DNA-damaging agents, and secondary metabolism were explored in the medically and industrially important fungus *A. terreus*. 

### 2.2. Deletion and Complementation of hstD in A. terreus

Two *A. terreus* wild-type strains, RA2905 and Mj106, were used to explore the function of HstD in *A. terreus*. Mj106 is a high producer of lovastatin, while RA2905 has been demonstrated to produce a high level of terrein [17]. To characterize the function of HstD, the deletion mutant (Δ*hstD*) was constructed by homologous recombination in these two *A. terreus* strains. To confirm whether the phenotype observed in the deletion mutants was caused by the disruption of *hstD*, the *hstD* gene was re-introduced into the Δ*hstD* mutants derived from RA2905 and Mj106, respectively, to obtain the complementation strains. The deletion mutant and complementation strains were verified by both diagnostic PCR and RT-PCR (Figure S1).

### 2.3. HstD Is Important for Mycelial Growth, Conidial Germination and Formation

**Figure 2:**
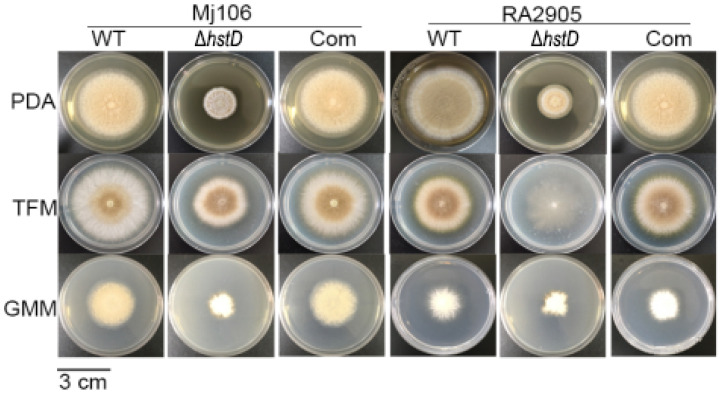
Mycelial growth and conidiation of *A. terreus* strains. Mycelial growth on PDA, TFM, and GMM, and photographs were taken 6 d after inoculation. All colonies were initiated by spotting 1 μL aliquots of a 10^6^ conidia mL^−1^ suspension.

**Figure 3:**
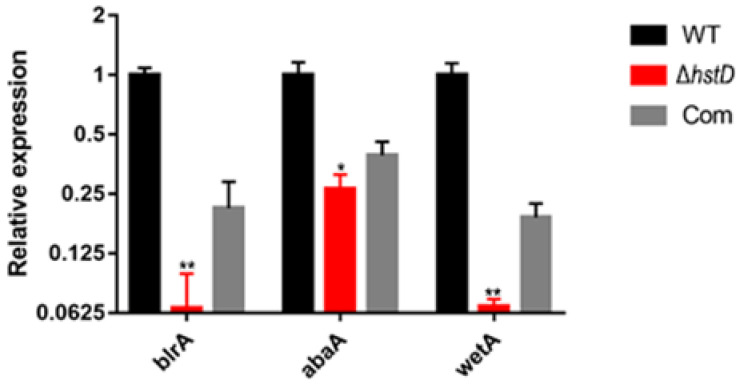
Analysis of expression levels of genes for conidiation. qPCR analysis of *blrA*, *abaA,* and *wetA* expression levels in *hstD* mutants and WT strains cultured in the PDB medium at 30 °C, 150 rpm for 48 h, with the expression level of actin as the internal control. All results were represented as the average ± SE of triplicate samples. Value 1 represents the expression level of *blrA* in the WT strain, and relative expression levels of *abaA* and *wetA* were normalized to the expression of *brlA* in WT. * *p* ≤ 0.05, ** *p* ≤ 0.01.

**Figure 4:**
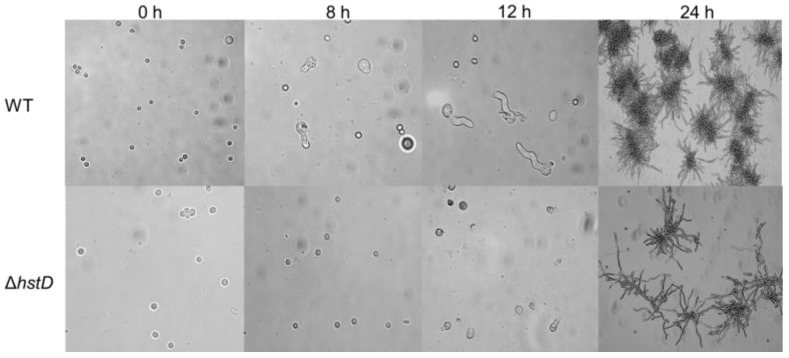
Conidial germination of *A. terreus* strains. Conidia (10^5^ mL^−1^) cultured in PDB broth with photographs taken at 0 h, 8 h, 12 h, and 24 h after inoculation.

Compared to wild-type and complementation strains, both Δ*hstD* mutants displayed significantly reduced mycelia growth, with a less pronounced effect on TFM agar than GMM or PDA agars (Figure 2). Additionally, the quantitative analyses of conidia numbers showed that asexual spore production in the Δ*hstD* mutant was dramatically decreased compared to WT and complemented strains. When grown on PDA or GMM media, almost no candida could be observed in Δ*hstD* from the Mj106 background, and the Δ*hstD* mutant produced only 30% conidia on the TFM agar compared with WT or complementation. Additionally, the conidia of Δ*hstD* from the RA2905 background were greatly reduced when cultured on PDA (10 folds), TFM (50 folds), and GMM (almost no conidia) agars. Accordingly, expression levels of the key asexual regulator genes *abaA*, *brlA*, and *wetA* were significantly downregulated in *hstD* deletion mutants compared to the WT and complementation (Figure 3). It was also shown that the conidia of the WT strain germinated at 6 h after inoculation and formed branched and interlaced mycelia at 24 h; however, the *hstD* mutant germinated at 12 h (Figure 4). Additionally, the mycelia of Δ*hstD* showed a reduction in branching compared to that of WT (Figure 4). These results suggest that HstD plays a crucial role in fungal normal growth, proper conidiation, and punctual germination in *A. terreus*.

### 2.4. HstD Is Involved in the Regulation of Secondary Metabolism

**Figure 5:**
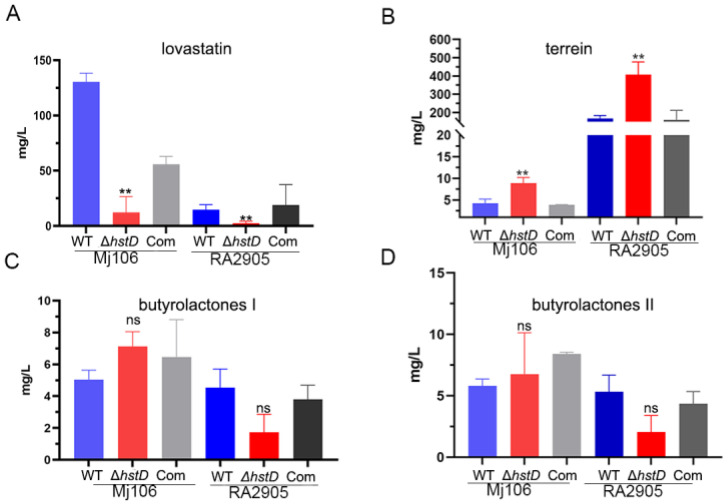
Biosynthesis of secondary metabolites, including lovastatin, terrain, and butyrolactones I and II in *A. terreus* strains. (**A**) Lovastatin, (**B**) Terrein, (**C**) Butyrolactones, I(**D**) Butyrolactones II. These data represent the mean value from three biological repeats; one-way ANOVA differences were considered significant when the *p*-value was ≤0.01 (**) and ns mean not significant.

Previous studies have shown that sirtuins can regulate secondary metabolism in various fungi [14]. Therefore, the effects of *hstD* deletion on secondary metabolism in *A. terreus* were investigated. The production of four major secondary metabolites in the *hstD* deletion mutant, complementation, and WT strains was analyzed using LC-MS/MS (Figure 5). The results indicate that lovastatin was the major producer of Mj106, while terrein was the primary producer of RA2905, which is consistent with our previous findings [17]. Clearly, the Δ*hstD* mutant derived from Mj106 produced a significantly reduced titer of lovastatin (12.1 ± 14.4 mg/L) compared to that of WT (130.5 ± 7.8 mg/L) or complementation (55.8 ± 7.2 mg/L) (Figure 5A). Furthermore, the deletion of *hstD* in RA2905 resulted in the almost undetectable production of lovastatin (Figure 5B). However, the Δ*hstD* mutant from RA2905 synthesized threefold more terrein (408.5 ± 48.5 mg/L) than that of the WT strain (168.5 ± 10.5 mg/L) or complementation strain (160.0 ± 37.1 mg/L) (Figure 5B). Consistently, the loss of HstD in Mj106 also increased terrein production; however, it was still lower than that of the RA2905 wild type strain and its derivatives. On the contrary, the loss of HstD seemed to have little effect on the biosynthesis of butyrolactone I or butyrolactone II (Figure 5C,D).

**Figure 6:**
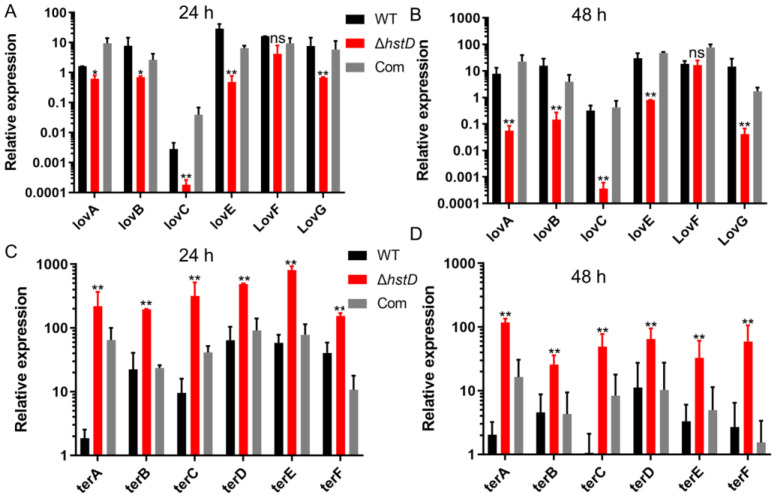
Analysis of expression levels of lovastatin and terrein biosynthetic genes. qPCR analysis of *lovA*-*lovG* and *terA*-*terF* expression levels in *hstD* mutants and WT strains cultured in LFM or TFM at 30°C, 150 rpm for 72 h, with the expression level of actin as the internal control. All results were represented as the average ± SE of triplicate samples. (**A**,**B**) values of 1 represent the expression level of *lovA* in the WT strain and the relative expression level of *lovA* mutants. Other lov genes in all strains were normalized to the expression of *lovA* of WT. (**C**,**D**) values of 1 represent the expression level of *terA* in the WT strain, and the expression level of *terA* in mutants and other ter genes in all strains were normalized to the expression of *terA* in WT. * *p* ≤ 0.05,** *p* ≤ 0.01, ns: not significant.

The biosynthesis of lovastatin involves two polyketide synthases, LovB and LovF, one P450 monooxygenase (LovA), one thioesterase (LovG), one reductase (LovC), and one incluster transcription factor LovE [1]. Accordingly, our results revealed that the expression of genes in the lov gene cluster, including *lovA*, *lovB*, *lovC*, *lovE*, *lovF,* and *lovG*, was greatly downregulated in the *hstD* disruption mutant (Figure 6A). For terrein biosynthesis, polyketide synthase (TerA), the multidomain protein (TerB), two FAD monoogenases (TerC and TerD), one multicopper oxidase (TerE), and one transcription factor were involved [18]. By contrast, terrein biosynthetic genes, including *terA-terF,* were significantly upregulated in the Δ*hstD* mutant compared with WT or the complementation strains (Figure 6B). These results collectively suggest that HstD positively regulated lovastatin biosynthesis but negatively regulated terrein biosynthesis, which has less effect on bactyrolactone synthesis.

### 2.5. Loss of HstD Does Not Sensitize A. terreus to DNA Damage Agents

**Figure 7:**
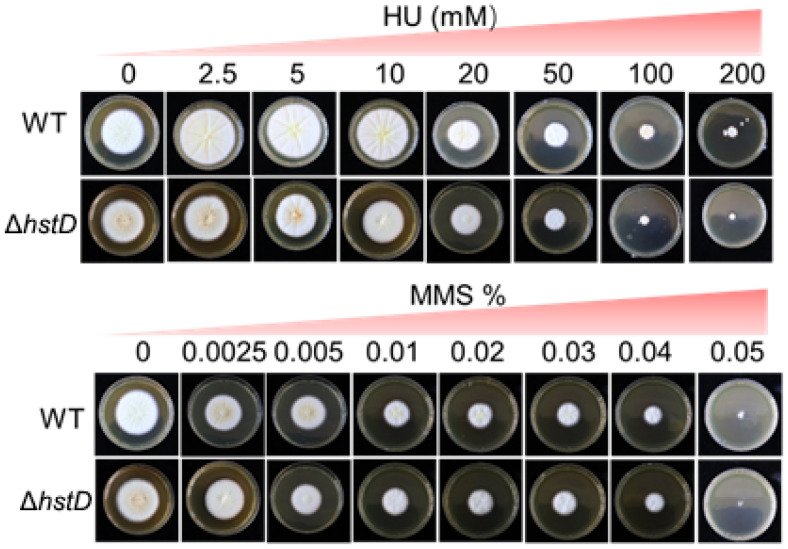
Effects of hydroxyurea and methyl methanesulfonate on the growth of *A. terreus* strains. Mycelia growth on PDA supplemented with or without HU or MMS, and photographs were taken 4 d after inoculation. All colonies were initiated by spotting 1 μL aliquots of a 10^6^ mL^−1^ conidia suspension.

It is known that sirtuins were used by fungi to regulate genome stability for their response to nutrient availability. It has been reported that the loss of sirtuin results in increased sensitivity to DNA damage agents in yeast [14]. Here, the *hstD* deletion mutant response to DNA damage agents, including hydroxyurea (HU) and methyl methanesulfonate (MMS), was examined. The results show that no effect on fungal growth caused by deleting *hstD* was observed under HU or MMS stress (Figure 7). Additionally, conidial development and the germination of Δ*hstD* was the same as in WT under HU or MMS stress. These results demonstrate that the loss of HstD could not sensitize *A. terreus* cells to DNA damage agents, which was different from their observation in yeast cells [19,20,21].

### 2.6. HstD Catalyzes the Deacetylation of Both Histone and Non-Histone Substances in A. terreus

**Figure 8:**
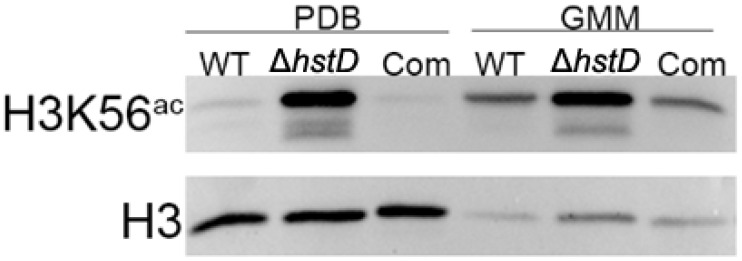
Assay acetylation levels of H3K56 and H3 of *A. terreus* strains. Conidia (10^−5^ mL^−1^) cultured in PDB and GMM broth and cultured for 48 h after inoculation to extract whole proteins. The protein level of histone H3 was used as an internal control.

It is well known that Hst3 and Hst4 (homologs of HstD) are responsible for removing the acetylation of K56 for histone H3 in yeast [22]. It has been proposed that HstD performed a similar function in *A. terreus*. As expected, our Western blot results showed that the acetylation level of H3K56 was significantly increased in the *hstD* deletion mutant when compared to WT or complementation strains, regardless of the culture condition (Figure 8). 

**Figure 9:**
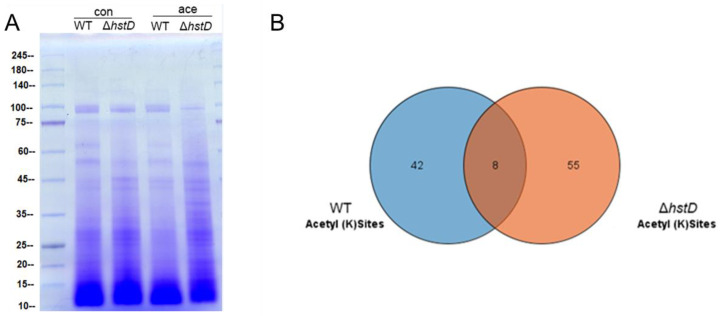
Analysis of acetylome and HstD-specific acetylome in *A. terreus*. Conidia (10^−5^ mL^−1^) cultured in LFM broth and cultured for 72 h after inoculation to extract and enrich acetylated peptides. (**A**) SDS-PAGE analysis of whole cell proteins (con) and acetylated proteins (ace) between Δ*hstD* and WT (**B**). Veen diagram of shared and specific acetyl sites of Δ*hstD* and WT.

It was recently reported that cellular substrates of lysine acetyltransferases or deacetylases were not restricted to histones: a large number of non-histone substrates were identified and involved in a variety of biological processes [23,24]. To comprehensively profile the substrates of HstD, we performed and compared the lysine acetylome of the *hstD* deletion mutant and wild-type strains using immune-affinity-based purification integrated with high-resolution mass spectrometry (Figure 9 and Tables S3 and S4). We observed that the disruption of HstD resulted in a significantly different acetylome compared with that of WT (Figure 9A). By LC-MS/MS analysis, a total of 63 acetylated peptides were identified in Δ*hstD*, which was greater than that of WT (50). Eight acetylated peptides were shared by both Δ*hstD* and WT, including H3K23, H3K56, glutaredoxin (K160), serine/threonine-protein phosphatase (K651), malate dehydrogenase (K318 and K340), the reduced viability upon starvation protein, inorganic diphosphatase, and uncharacterized proteins (K92 and K96). In addition, other proteins with acetylated peptides in the wild type of *A. terreus* Mj106 include histone H2A and H2B, Zn2Cys6 transcription factors, kinases, and metabolic enzymes (Table S3). Interestingly, 55 acetylated peptides (belonging to 42 proteins) were uniquely enriched in the acetylome of Δ*hstD*. These acetylation proteins were mainly classified into uncharacteristic biological functions or metabolic enzymes. Notably, seven proteins with two acetylated lysine sites were uniquely detected in Δ*hstD*, including adomet-dependent rRNA methyltransferase SPB1, a major facilitator superfamily protein, and five unknown proteins (Table S4), implying that these targets partake in the roles of fungal growth, development, or secondary metabolism.

## 3. Discussion

Sirtuins are NAD+-dependent deacetylases that are well-known for their ability to catalyze the deacetylation of the histone, leading to gene expression repression and stabilizing the genome [14]. Recently, their regulatory roles in NAD homeostasis, including primary and secondary metabolism, development, and the virulence of pathogenic fungi, have been gradually unveiled. However, a variety of secondary metabolites are simultaneously produced in any one of the filamentous fungi; therefore, the regulatory mechanism mediated by the deacetylase of sirtuins and its targets is complex and largely unknown. For example, sirtuin negatively regulates the biosynthesis of austinol, dehydroaustinol, and sterigmatocystin but positively regulates other secondary metabolites by the deacetylation at H4K16 [25]. It is noteworthy that sirtuin has not been identified in *A. terreus* or most other filamentous fungi. Previous results showed that nicotinamide, a potent inhibitor of sirtuins, significantly changed the metabolite profile of filamentous fungi [26]. Additionally, the effect was observed in *A. terreus* RA2905. The results observed here further demonstrate that sirtuin HstD is an important regulator of secondary metabolites, including lovastatin and terrein, which could be targeted for genetic engineering to improve the yield of these valuable compounds. 

In fungi, the high acetylation of H3K56 facilitates histone-associated DNA during the S phase, while its low acetylation dissociates DNA and histone complexes during the G2/M phase [21]. Hst3 or/and Hst4 (homologs of HstD, only one in some fungi) function as the major deacetylases of H3K56. Therefore, an abnormal (too high or low) acetylation level of H3K56 renders fungi more susceptible to genotoxic agents. Indeed, the disruption of Hst3 or Hst4 (as well as the double mutant) in *S. cerevisiae* decreases the survival of cells exposed to the DNA damage agents of methyl methanesulfonate (MMS) and hydroxyurea (HU) [20]. In *Schizosaccharomyces pombe*, however, Δ*hst4* cells only show increased sensibility to MMS but not HU [19]. Notably, the loss of HstD did not sensitize cells of *A. terreus* to both DNA damage agents, even though HstD deletion also resulted in the elevated acetylation level of H3K56 as in *S. pombe* and *S. cerevisiae*. There were two speculations to explain this observation. First, as a filamentous fungus, *A. terreus* had a stronger ability to tolerate MMS or HU and a faster recovery rate from the resultant DNA damage than yeast. Therefore, it was difficult to observe the difference between the mutant and WT by the colony phenotype. This speculation was partially supported by the fact that higher DNA damage agents were required to completely inhibit the growth of *A. terreus* (above 0.05% MMS or 200 mM) compared to *S. pombe* or *S. cerevisiae* (about 0.01% MMS or 100 mM). Second, from the perspective of molecular mechanisms, both MMS and HU cause the stalling of the replication fork. Then, DNA damage was repaired by several pathways (homologous recombination and non-homologous end joining). It was speculated that a more efficient DNA repair process was inspired in the Δ*hstD* cell of *A. terreus* than in the Δ*hst4* of yeasts. This speculation was partially supported by the fact that a number of enzymes involved in DNA repair were significantly changed after the loss of HstD in *A. terreus*. For example, the DNA repair proteins RAD9, CRT10, and the sex-determining protein Fem-1 were specifically acetylated in Δ*hstD*. Considering that DNA repair is a complex process, further explorations of underlying and detailed mechanisms of their observation by genetic and biochemical analysis were required.

Sirtuins have been widely explored as histone deacetylases and their concomitant regulatory roles in yeast and other eukaryotic organisms. However, these enzymes also catalyze the deacetylation of non-histone targets to modulate cellular physiology and metabolism [27]. Together with our results, it was speculated that HstD regulated fungal growth, conidiation, and secondary metabolism via two independent mechanisms. One was epigenetic control. First, our WB results showed that HstD catalyzed the deacetylation of H3K56. Second, the observation of acetylated H3K27 specially enriched in *hstD* deletion mutants led to the identification of one novel histone target of HstD for the first time in fungi. Therefore, increased acetylation levels of these histone sites (H3K27 and H3K56) in the *hstD* mutant altered the expression of a set of genes involved in several cellular processes, including genome stability maintenance, fungal growth, conidiation, and the biosynthesis of metabolites [21,28,29]. An alternative regulatory mechanism is that HstD performs post-translational modifications of downstream regulators or enzymes. Actually, our proteome data demonstrated that the acetylation level of whole cell proteins was significantly increased in the Δ*hstD* mutant compared to WT, and 42 proteins with a variety of activities were specifically acetylated as a result of *hstD* deletion. For example, the 14-3-3 protein was acetylated in Δ*hstD*, and its homolog was reported to be a global regulator of vegetative growth, conidiation, and secondary metabolism in *A. flavus* [30]. Additionally, the protein phosphatase Ppt1 was also acetylated only in the mutant. Ppt1 has been demonstrated to play an important role in fungal polarized growth, conidiation, and DNA repair in *Metarhizium acridum* [31]. Notably, most novel substrates of HstD are uncharacterized proteins; therefore, their function in fungal growth, development, and secondary metabolism requires further clarification.

## 4. Materials and Methods

### 4.1. Bioinformatic Analysis

Sirtuin protein sequences of *S. cerevisiae* were obtained from the yeast database (SGD), and their homologs or paralogs in *A. terreus* and other filamentous fungi were downloaded from NCBI. The phylogenetic tree was generated using the neighbor-joining method based on MEGA software (version 6).

### 4.2. Strains and Media

The *A. terreus* wild-type strains RA2905 and Mj106, along with their mutants, were stored in 20% glycerol at −80 °C. For fungal growth and conidiation assays, fungal strains were grown on PDA (2% sea salt, potate extracts 2%, glucose 2% and 1.5% agar), GMM (Czapek’s salt and 2% sea salt), and TFM (2% sea salt, starch 4%, peptone 1%, glutamine 1% and 1.5% agar) media. For protein extraction, PDB and GMM broths were used to culture fungal strains. For secondary metabolite assays, LFM (2% sea salt, lactose 4%, KH2PO4 1.5%, peptone 0.5%, yeast extract 2% and 0.05% MgSO_4_) and TFM broths were used to induce *A. terreus* to produce lovastatin and terrein, respectively. Their responses to DNA-damaging agents were assessed by spotting conidial suspensions onto PDA media supplemented with either hydroxyurea (HU) (0–200 mM) or methyl methanesulfonate (MMS) (0–0.05%).

### 4.3. Construction of hstD Deleted and Complemented Strains

Gene deletion and complementation were performed as previously described with minor modifications [32]. Briefly, *Aspergillus terreus* RA2905 or Mj106 genomic DNA was used as a template to amplify 2000 bp upstream and downstream of the *hstD* as the homologous regions. The hygromycin resistance cassette (*hph*) amplified from pSilent-1, along with the upstream and downstream homologous sequences, were used to construct gene knockout cassettes using fusion PCR. The gel-purified gene knockout cassette was transformed into protoplasts of *A. terreus* RA2905 or Mj106 using the PEG-mediation method, and transformants were screened on the plate (200 μg/mL hygromycin) to obtain genetically stable gene knockout strains. The expression cassette for *hstD* with its native promoter was amplified from RA2905 or MJ106 and then transformed into the *hstD* mutant along with the pTR1 plasmid (containing the selectable marker gene *ptrA*). The validation of the mutant was undertaken for gene deletion or complementation by diagnostic PCR. All primers used here are listed in Table S1, and all strains described here are listed in Table S2.

### 4.4. Histone Acetylation Detection

The acetyl-histone H3 (Lys56) and histone H3 antibodies used in this study were purchased from Cell Signaling Technology (Danvers, MA; Catalog No.: #4243) and Abcam (Shanghai, China; Catalog No.: ab1791), respectively. *A. terreus* spores (10^6^ mL^−1^) were inoculated in Czapek–Dox or PDB media and incubated for 2 d with shaking at 150 r. p. m. at 30 °C. Mycelia were collected and ground under liquid nitrogen. The whole-cell protein was extracted using a RIPA lysis buffer (Beyotime, Shanghai, China), and the protein concentration was determined using the BCA Protein Assay Kit (Sangon, Shanghai, China). Proteins (30 μg) were separated on 12% SDS-PAGE and transferred to PVDF membranes (Sangon, Shanghai, China). Primary anti-H3K56 or H3 was added at a dilution of 1:2000. The anti-rabbit secondary antibody conjugated to horseradish peroxidase was added at a dilution of 1:5000. 

### 4.5. Secondary Metabolite Analysis

The lovastatin standard was purchased from Sorlarbio (Beijing, China), and the terrein standard was isolated previously [15]. To quantify the production of lovastatin and terrein, fungal conidia were pre-cultured in PDB media for 24 h and shaken at 150 rpm at 30 °C. Then, an aliquot of mycelia (0.5 g) was transferred into LFM and TFM media for fermentation (150 r. p. m. at 30 °C) for an additional 10 d. These cultures were extracted with ethyl acetate and evaporated to remove the ethyl acetate. The concentrations of lovastatin and terrein were separated and determined by UPLC-MS/MS on ACQUITY UPLC H-Class Plus XEVO TQ-S micro equipment (Waters, Milford, CT, USA). Experimental conditions were as follows: compounds were separated on an RD C18 chromatography column (4.6 mm × 100 mm, 3 μm) and eluted with an acetonitrile–water (formic acid 0.1%) gradient with a flow rate of 0.5 mL/min. The detection wavelengths of lovastatin and terrein were 240 nm and 280 nm, respectively. Then, the content of these two compounds in the fermentation broth was calculated with a standard curve. One-way ANOVA differences were considered significant when the *p*-value was ≤0.05 (*) and the *p*-value was ≤0.01 (**).

### 4.6. RNA Extract, cDNA Synthesis, and Quantitative Real-Time-PCR

Conidia (10^6^ mL^−1^) were inoculated into PDB broth and pre-cultured for 24 h. The mycelia were collected and then transferred into fresh LFM or TFM broth for culture at 28 °C for 48 h. The mycelia were collected via filtering and then frozen in liquid nitrogen and stored under −80 °C conditions. RNA extraction and cDNA synthesis were performed using the TRizol agent and RNA purification kit (Transgen, Beijing, China). Three replicates of cDNA samples (three replicates) were used as templates to quantify gene transcript levels in the cultures of each strain through qPCR with paired primers (Table S1) under the action of SYBR® Premix Ex TaqTM (Takara, Dalian, China). The expression of the actin gene was used as an internal control.

### 4.7. Lysine Acetylome

Conidia (10^6^ mL^−1^) were inoculated into LFM broth and cultured at 28 °C for 72 h. The mycelia were collected via filtering, were frozen in liquid nitrogen, and stored under −80 °C conditions. Whole-cell proteins were extracted by grinding and centrifuging. Trypsin digestion and the alkylation of protein concentration solutions were performed, as previously described [16]. Tryptic peptides were dissolved into the NETN buffer (100 mM NaCl, 50 mM Tris-HCl, 0.5% NP-40, 1 mM EDTA, pH 8.0) and incubated with pan anti-acetyl antibody beads (PTM Biolabs, Chicago, IL, USA). The beads were washed with an NETN buffer 43 times, eluted by 0.1% TFA, and then vacuum-dried to obtain acetylated peptides for LC-MS/MS analysis. 

Peptides were separated using a reversed-phase trap column (75 µm*150 mm, 3 µm, Dr. Maisch GmbH, Ammerbuch-Entringen, Germany) directly on an EASY-nLC 1000 UPLC system at a flow rate of 300 nL/min in the solvent containing 0.1% FA in 98% ACN and using a gradient concentration of 3–5% (2 min) and 5–80% (42 min). The Q-Exactive Plus mass spectrometer (Thermo Fisher Scientific, Waltham, MA, USA) was used to detect the acquired peptides. Ion fragments were detected by Orbitrap at a 17,500 resolution. A data-dependent procedure was applied with 10 MS/MS scans after 1 MS scan for the top 10 precursor ions above a threshold ion count of 2E4, with 10.0 s of dynamic exclusion. The *m/z* scan range was set to 350 to 1800. The MS/MS results were searched against the *A. terreus* database (http://fungi.ensembl.org/Aspergillus_terreus/, accessed on 23 May 2023) and linked to the reverse decoy database using MaxQuant(v1.4.1.2).

## Data Availability

All data generated and analyzed during this study were included in this manuscript and the Supplementary Materials.

## Supplementary Materials

The following supporting information can be downloaded at: https://www.mdpi.com/article/10.3390/ijms241612569/s1.
