# Supplementary material for: The Histone Deacetylase HstD Regulates Fungal Growth, Development and Secondary Metabolite Biosynthesis in Aspergillus terreus
Source: Int J Mol Sci. 2023 Aug 8;24(16):12569. doi: 10.3390/ijms241612569 (PMC10454297; doi:10.3390/ijms241612569)
Supplement: Supplementary file 1 [file ijms-24-12569-s001.zip › Figure S1.pdf]

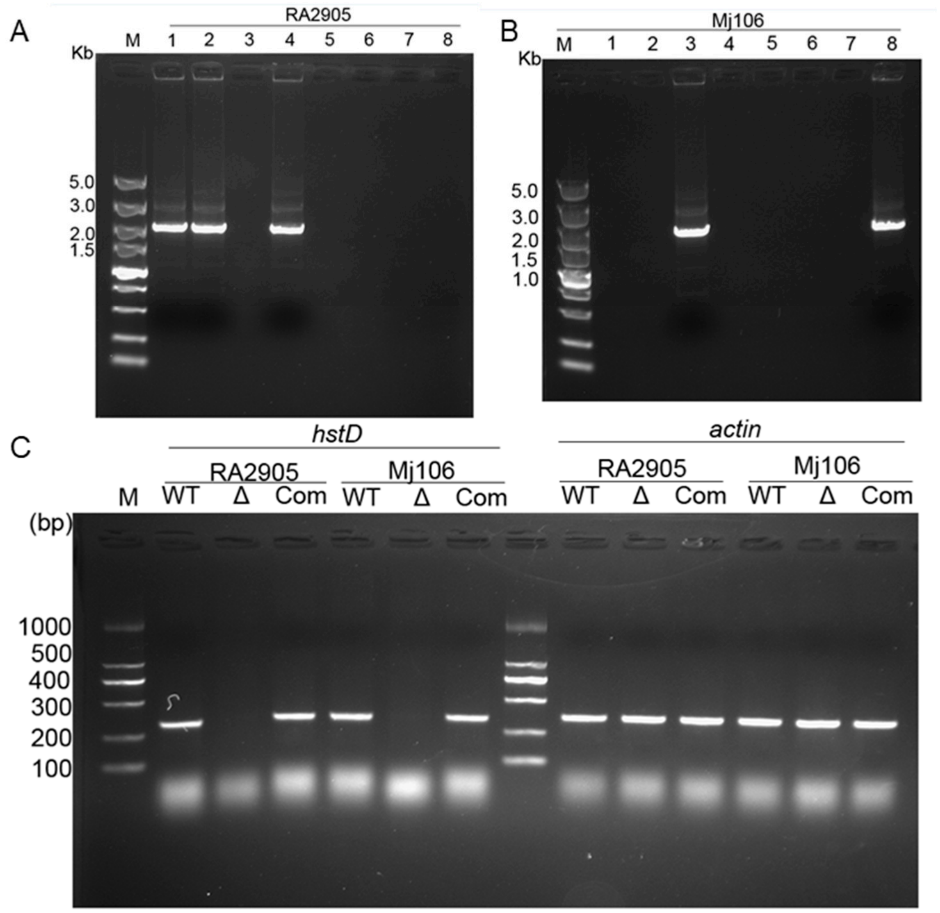

Figure S1 Diagnostic PCR and RT-PCR verification of *hstD* deletion and complementation.

(A) Diagnostic PCR verification of *hstD* deletion in RA2905 background by using hph-specific primers

(B) Diagnostic PCR verification of *hstD* deletion inMj106 background by using hph-specific primers

(C) RT-PCR verification of *hstD* deletion and complementation with *actin* gene as the control.
