# Supplementary material for: The Histone Deacetylase HstD Regulates Fungal Growth, Development and Secondary Metabolite Biosynthesis in Aspergillus terreus
Source: Int J Mol Sci. 2023 Aug 8;24(16):12569. doi: 10.3390/ijms241612569 (PMC10454297; doi:10.3390/ijms241612569)
Supplement: Supplementary file 1 [file ijms-24-12569-s001.zip › Table S1.pdf]

Primers used in the study

Name	Sequence (5`-3`)	Usage
HphF	CGACGTTAACTGATATTGAA	knock out
HphR	CAACCCAGGGCTGGTGACGG	knock out
HstDF1	GCCGAGTTAGTTTGGTTTGT	knock out
HstDR1	CCACGACCGAATCACTGCTA	knock out
HstDF2	CCACGGAGCAGAATAGGAGC	knock out or complementation
HstDR2	TCTCGCACGGTCTACGCTTT	knock out or complementation
HstDhphF	AAATTCCGTCACCAGCCCTGGGTTGTGTCACGATTTTACACTGCA	knock out
HstDhphR	AAATTCCGTCACCAGCCCTGGGTTGACACAAGCCAATGCACCCTC	knock out
HstDRTF	GCTCCACTGATGGGCTATTT	Verification
HstDRTR	GACCACCTTGTCGAGACTGC	Verification
wetARTF	CATTGTCAAGCGATTGTGGC	qPCR
wetARTR	CGGTTCTCGGTAGGGCAGTT	qPCR
brlARTF	CCTTCAAGACGCATACTCCG	qPCR
brlARTR	TTTCATGTGGCGCTTCAGGT	qPCR
abaARTF	ACACGGGCAAGGATGGGGA	qPCR
abaARTR	TTCCATGGAGGTCCGCCAC	qPCR
lovARTF	GGAGTCACAGCGAGTCAA	qPCR
lovARTR	CCTTAGAGGCAAAGAACC	qPCR
lovBRTF	CGGCAGATGGAGACAAAG	qPCR
lovBRTR	GGCGAGGACGGTTACAAT	qPCR
lovCRTF	GCAAATGCTACGCCTGTC	qPCR
lovCRTR	TCGGTCGTGACCATCTTA	qPCR
lovERTF	GCCCTTCGAGTCTACGGT	qPCR
lovERTR	TTAGCCAGTTTGCCCACA	qPCR
lovFRTF	GGATGGCAGGATGTGATG	qPCR
lovFRTR	GAAGCCCGAAAGTGAAGA	qPCR
TerARTF	AGCAGCAAGTCATACAGCAA	qPCR
TerARTR	AACTCCTCGCATAACAAAGC	qPCR
TerBRTF	CAAGGATTGAAGGCGGGATC	qPCR
TerBRTR	AGAAGTTTCGGGCACAGGTT	qPCR
TerCRTF	GCATCTGCAAAGCCTCCTGT	qPCR
TerCRTR	CTCTGGCGTGTCTCATACCG	qPCR
TerDRTF	TCAGGCTGGAGACAGTAATC	qPCR
TerDRTR	AACGCATCTGGTATCTGGTC	qPCR
TerERTF	TGGTGCCTCCCGAGAAACAG	qPCR
TerERTR	CTCGCCGACCTTGGCTACAT	qPCR
TerFRTF	TACGACCAGGAAGGAACAGT	qPCR
TerFRTR	GAGCCGATAGTAGCAGAAGC	qPCR
ActinF	GGTTACACCTTCTCCACCAC	qPCR
ActinR	CCTTACGGACATCAACATCA	qPCR
