# Supplementary material for: The Histone Deacetylase HstD Regulates Fungal Growth, Development and Secondary Metabolite Biosynthesis in Aspergillus terreus
Source: Int J Mol Sci. 2023 Aug 8;24(16):12569. doi: 10.3390/ijms241612569 (PMC10454297; doi:10.3390/ijms241612569)
Supplement: Supplementary file 1 [file ijms-24-12569-s001.zip › Table S2.pdf]

Name	Genetic type	resource
RA2905	wild type	[14]
RAΔ	*ΔhstD*::*hph*	This study
RACom	*ΔhstD*::*hph-hstD*+*ptrA*	This study
Mj106	wild type	*Acanthus ilicifolius*
MjΔ	*ΔhstD*::*hph*	This study
MjCom	*ΔhstD*::*hph-hstD*+*ptrA*	This study
